# Expectations regarding school decreases emotional distress among college students in Western China: the buffering role of physical exercises

**DOI:** 10.3389/fpubh.2024.1412199

**Published:** 2024-11-06

**Authors:** Di Su, Lina Huang, Helin Zou, Lulu Zhang, Yi Feng

**Affiliations:** ^1^Mental Health Counselling Centre, Ningxia University, Yinchuan, China; ^2^Department of Psychology, School of Social Sciences, Tsinghua University, Beijing, China; ^3^School of Sport Economics and Management, Central University of Finance and Economics, Beijing, China; ^4^School of Statistics and Mathematics, Central University of Finance and Economics, Beijing, China; ^5^Mental Health Education and Counseling Center, China University of Petroleum-Beijing at Karamay, Xinjiang, China; ^6^Mental Health Center, Central University of Finance and Economics, Beijing, China

**Keywords:** emotional distress, physical exercise, school belongingness, school exclusion, expectations regarding school

## Abstract

**Background:**

College students in Western China face unique economic, cultural, and educational environments, yet limited studies have specifically investigated the factors or interventions concerning emotional distress within this population.

**Aim:**

This study aimed to explore whether school belongingness mediates the relationship between expectations regarding school and emotional distress among college students in Western China, and whether physical exercise moderates this mediation.

**Methods:**

Employing a cross-sectional design, 1,063 college students in Xinjiang, China were recruited for this study. A self-administered electronic questionnaire assessed expectations regarding school, school belongingness, physical exercise, anxiety, and depression. Structural equation modeling was utilized to analyze mediating and moderating effects.

**Results:**

Expectations regarding school was negatively associated with emotional distress. School exclusion and school acceptance fully mediated the effect of expectations regarding school on emotional distress. Physical exercise moderated the mediating effect of school exclusion, but not that of school acceptance.

**Conclusion:**

Expectations regarding school and school belongingness, particularly the exclusion component, emerge as pivotal factors influencing emotional distress among college students in Western China. Furthermore, physical exercise presents itself as a promising targeted intervention for alleviating emotional distress within this demographic.

## 1 Introduction

Mental health among college students is a pressing issue in China (1), as evidenced by studies highlighting significant challenges, particularly regarding anxiety and depression (2, 3). Recent data indicates a significant rise in the prevalence of these mental health concerns (4), raising serious concern for the wellbeing of college students. Chinese students face multiple transitional challenges during college, including physical maturation, academic demands, adaptation to communal living, and planning for future careers (5, 6). These complex transitions underscore the importance of addressing mental health issues within the college population.

China’s expansive territory encompasses significant cultural and geographical distinctions between its eastern and western regions, leading to diverse lifestyles and values among its populace (7, 8). Educational institutions in Western China notably lag behind their counterparts in the Eastern China and Southern China, grappling with substantial disparities in educational resources (9–12). Many students choose to study in the more developed urban centers of Eastern and Southern China, as western universities are not their first choice. Consequently, students admitted to western universities typically have lower entrance exam scores and lack academic competitiveness, which further exacerbates the challenges they face during transitions and adaptations, compounded by the region’s weaker economic development (10, 12–14). As of June 2023, Western China hosts 763 general higher education institutions, comprising 27.06% of the whole nation (15). Despite this, most research endeavors have predominantly focused on participants from eastern or central colleges (3), with limited attention dedicated to the mental health status of college students in western institutions, which may be comparably more pronounced (3). Thus, it is imperative to investigate the mental health status and associated influencing factors among college students in Western China, acknowledging them as a distinct cohort deserving of targeted research efforts. This study aims to fill this research gap by being the first to explore the influence of expectations regarding school, belongingness, and physical exercise on emotional distress. Unlike research in more affluent regions, this study provides insights into the unique socio-economic disadvantages and limited educational opportunities faced by university students in Western China, and aims to explore the mechanism of how expectations regarding school affects their adaptation and mental health levels from a new perspective of psychological resilience in such contexts.

### 1.1 Expectations regarding school and emotional distress

When transitioning from high school to university, students often have certain expectations about their future university, which previous research has largely focused on in terms of educational expectation (5, 16–18). However, due to the requirement for Chinese university students to reside in on-campus dormitories, students’ expectations of school go beyond academics to include campus environment and school life. In current study, the term “expectations regarding school” is introduced to confer to students’ expectations about school life, academics, and other aspects of the student experience (19–23).

Based on the theory of psychological capital (24), students’ expectations regarding school reflect their optimistic outlook, influencing how they perceive their surroundings (25) and signaling increased hope for both the school environment and their future growth trajectory (25, 26). These attributes constitute pivotal factors in fostering happiness and positive developmental outcomes (27, 28), playing a critical role in enhancing the psychological adaptation of college students (29). Previous research has demonstrated its crucial role in mitigating emotional distress among individuals (30, 31).

The present study specifically delves into the influence of expectations regarding school on the emotional distress experienced by college students in Western China, a demographic confronted with pronounced challenges stemming from the region’s weak economic development (9). Within the socioecological framework of psychological resilience, facing the adverse circumstances in the western region of China, students’ expectations regarding school can serve as a protective mechanism and buffer, mitigating the negative effects of various stressors, including familial economic circumstances, academic and career pursuits, as well as public emergencies such as the COVID-19 pandemic (32–34). Consequently, we hypothesize that students’ expectations regarding school in Western China plays a pivotal role in their management of emotional distress.

### 1.2 Expectations regarding school and school belongingness

Students’ expectations significantly influence their adjustment to college life, serving as facilitators to successful integration (35, 36). For example, students anticipating a welcoming and inclusive college environment are more inclined to actively pursue social opportunities and engage with peers, consequently fostering heightened levels of school belongingness and mental well-being. School belongingness denotes a student’s perception of connection, acceptance, and inclusion within the educational setting (37, 38). It encompasses two dimensions: school acceptance, which refers to how welcomed, valued, and included students feel within their school community, and school exclusion, which is the feeling of being left out, rejected, or alienated (38–40). School belongingness addresses the basic psychological need among college students, offering a sense of purpose, significance, and value within the school community (40).

School belongingness may be influenced by various factors such as school climate, teacher-student relationships, and perceived support, which constitute key components of college students’ expectations regarding school (19, 37). Research has shown that students who hold higher expectations for their future tend to possess a stronger sense of school belonging (41). Furthermore, in accordance with the Expectancy–Value Theory, expectations regarding school can function as motivational drivers, inspiring college students to pursue and achieve their goals and accomplishments with proactive adaptive behaviors (42). Examples of such behaviors include active participation in extracurricular activities, fostering interpersonal connections, and maintaining a diligent approach to academics, which are believed to enhance a sense of school belonging among college students (41). Therefore, it is hypothesized that college students’ expectations regarding school may serve as a positive predictor of their sense of school belongingness.

### 1.3 School belongingness and emotional distress

Schools play a pivotal role in influencing the mental wellbeing of young individuals by nurturing the development of social–emotional skills, fostering inclusive and safe environments, and cultivating a sense of community (43). A sense of belonging in the school environment serves as a significant predictor of students’ wellbeing (44, 45), positive affect (45), and prosocial behavior (46). According to the self-determination theory, individuals have an inherent desire of belonging where they feel valued and cared for, which contributes to their overall mental health and mitigates negative emotions (47). Conversely, a lack of acceptance or feelings of exclusion within the school community can negatively impact psychological, behavioral, and social outcomes. These repercussions may manifest as feelings of loneliness (44), engagement in violent or risky sexual behavior, and involvement in substance abuse (48). These findings suggest the necessity to explore the potential protective influence of school belongingness on emotional distress among college students in Western China.

Among college students, anxiety and depression represent the most prevalent mental health concerns (49). Existing literature suggests that school belongingness, encompassing feelings of acceptance and exclusion, can serve as a buffer against symptoms of depression and anxiety (50–52). Regarding anxiety, students who feel accepted within their academic community tend to receive more tangible support, thereby reducing apprehension related to life stressors, academic pressures, and financial burdens (53). Conversely, students who experience physical and emotional exclusion may encounter social anxiety (54) and engage in rumination, exacerbating their feelings of anxiety (55). With regard to depression, receiving acceptance and support within the school environment fosters positive feedback, which, in turn, promotes positive emotions and diminishes the risk of depression (56, 57). Conversely, feelings of self-negation and low self-esteem stemming from exclusion may heighten the risk of depression (58).

In summary, the impact of expectations regarding school on emotional distress among college students may be mediated by their sense of school belongingness. This mediation process can be delineated into two parallel pathways: acceptance and exclusion. We seek to integrate this mediation process within the framework of uncertainty. Research has demonstrated that individuals form expectations about uncertain future events from early stages of development (59). These expectations often entail a degree of uncertainty (60). According to the uncertainty-identity theory, individuals confronted with uncertainty tend to exhibit heightened group identification and favoritism towards their in-group (61), which serves to mitigate subsequent uncertainty and alleviate associated negative affect and emotional distress (62, 63). As a result, college students with higher level of expectations regarding school may encounter greater uncertainty. Consequently, they may engage in behaviors such as active participation in extracurricular activities, fostering identification with the school, and integrating with peers. Thus, we hypothesize that, as a protective mechanism, expectations regarding school may enhance individuals’ sense of acceptance and mitigate the risk of exclusion, thereby reducing emotional distress associated with uncertainty.

### 1.4 Physical exercise and emotional distress

In terms of the impact on emotional distress, school exclusion emerges as the more significant aspect of school belongingness (51). We suppose that the discrepancy in school belongingness, particularly concerning school exclusion, and their effects on mental health may be buffered by certain factors, such as physical exercise, which is commonly acknowledged as a means of fostering personal psychological resilience (64). Physical exercise benefits individuals confronting adversity, aligning with the framework of psychological resilience from a socioecological perspective (65). Research indicates that physical exercise exerts a positive effect on alleviating depression and anxiety among Chinese university students (66) and can diminish social problems, thought problems, and attention problems among adolescents (67).

The moderating effects of physical exercise on emotional distress resulting from school exclusion can be delineated into psychological and neurophysiological mechanisms. Firstly, concerning psychological mechanisms, engaging in physical activities can bolster individuals’ self-esteem and self-efficacy, enabling them to rebound from exclusion and serving as a protective factor for mental health when facing ostracism (68). Additionally, involvement in physical exercise may divert excluded students’ attention away from depression thoughts, discomfort and painful emotions (69, 70).

Secondly, the neurophysiological effects of physical exercise also contribute to its moderating influence. Extensive research indicates that chronic physical exercise is a promising strategy for enhancing critical executive functions such as inhibitory control and working memory (71), by augmenting the structure and function of the brain (72), particularly the prefrontal cortex, which plays a pivotal role in emotion regulation (73). Furthermore, acute exercise triggers activation in the medial prefrontal cortex and medial temporal lobe, associated with episodic memory function, a crucial cognitive ability for maintaining overall wellbeing (74, 75). These enhancements in cognitive and emotional function resulting from physical activity may aid students in better appraising exclusionary events, recovering from negative feelings, and averting long-term emotional distress (76).

Consequently, we posit that physical exercise is likely to moderate the effects of expectations regarding school on emotional distress, while also serving as a boundary condition for the mediating effects of school exclusion to manifest.

### 1.5 Aim of this study

This study aims to assess the influence of expectations regarding school and school belongingness on emotional distress among college students in Western China, while also to explore the moderating role of physical exercise. Specifically, for college students in Western China who face socioeconomic disadvantages and limited educational opportunities, how does expectations regarding school function to alleviate emotional distress? We hypothesize that school belongingness (particularly school exclusion) plays an important mediating role in this process. Another key research question is what role physical exercise plays in helping college students in Western China adapt to their school environment and regulate their emotions. We hypothesize that physical exercise will moderate the effects of expectations regarding school and school exclusion on emotional distress.

## 2 Methods

### 2.1 Participants and procedure

This study utilized a cross-sectional design by convenience sampling and snowball sampling. A survey was conducted from July 25th to October 5th in Xinjiang, Western China. A total of 1,063 college students were recruited and completed an online questionnaire administered through Credamo, a research platform in China. Some of the samples were collected using a convenience sampling strategy through the Credamo platform, which allows for the specification of the target population through tagging. The platform randomly distributed the survey to ensure the randomness of the data. In addition, we collected other samples by sending a QR code to university teachers in Xinjiang. The teachers distributed this QR code to undergraduate and graduate students, and conducted consequent snowball sampling. Each participant was allowed to complete the survey only once. Prior to participation, respondents were briefed on the study’s objectives and their right to withdraw at any point. Individual informed consent was obtained from each participant on the initial page of the survey. This study involving human participants was following the Declaration of Helsinki’s ethical standards and were reviewed and approved by the Research Ethics Review Committee of Central University of Finance and Economics, China.

The study implemented two exclusion criteria to maintain the quality of the sample: (a) An attention check was incorporated into the survey by including an additional question instructing participants to select a specific response (e.g., “Please select ‘ *seldom* ’ directly”). Participants who did not comply with this instruction were excluded from the analysis. (b) Individuals who self-reported a history of emotional distress were also excluded from participation in the study. These criteria excluded 41 participants, with the final sample comprising of 1,019 participants. The response rate was 95.86%.

### 2.2 Materials

The measurement includes demographic information and main variables in this study. Detailed measurement for all main variables was in Supplementary Table S1.

#### 2.2.1 Demographic variables

The questionnaire comprised several scales alongside demographic inquiries. The demographic measures encompassed age, sex, ethnicity, educational level, place of birth, and whether respondents were the only child in their family.

#### 2.2.2 Expectations regarding school

Expectations regarding school was assessed by four self-designed questions, i.e., “How were your expectations of campus environment?,” “How were your expectations of campus life?,” “How were your expectations of academic experience?” and “How were your expectations of extracurricular activities?” (see detailed scale development procedure for the measurement in Supplementary material). Participants were asked to recall what they thought before entering the school, and to answer the questions accordingly. Responses were rated on a 5-point scale, ranging from “*very high*” to “*very low*.” The scores from the four items were aggregated to create a composite score of expectations regarding school, with higher scores indicating higher expectations.

#### 2.2.3 School belongingness

School belongingness was assessed using the School Belongingness Scale (SBS) (40), which comprises 10 items divided into two subfactors: school exclusion and school acceptance. These subfactors were measured using the School Exclusion Scale (SES) and School Acceptance Scale (SAS) respectively, each consisting of five items. Participants rated their responses for each item on a four-point Likert scale (1 = a*lmost never*, 2 = *rarely*, 3 = *sometimes*, 4 = *almost always*). The SBS demonstrated high internal reliability coefficients in this study (Cronbach’s *α* = 0.871), while both the SES (Cronbach’s *α* = 0.832) and SAS (Cronbach’s *α* = 0.858) exhibited good internal reliability. Additionally, a significant negative correlation was observed between SES and SAS (*r* = −0.506, *p* < 0.001).

#### 2.2.4 Emotional distress

Emotional distress is a multifaceted construct that encompasses various emotional states, often characterized by symptoms of anxiety and depression (77). Previous research suggested that anxiety and depressive diagnoses frequently coincide, with their symptoms demonstrating a high degree of correlation (78, 79). Consequently, in the present study, emotional distress was evaluated using two indicators: anxiety and depressive symptoms.

Anxiety symptoms were assessed using the 7-item Generalized Anxiety Disorder Scale (GAD-7) (80), a self-reporting screening scale that has been validated in China (81). Participants reported on their anxiety symptoms over the past 2 weeks using a 4-point scale (1 = *not at all*, 2 = *several days*, 3 = *more than half the days*, 4 = *nearly every day*). Sample statements included “not being able to stop or control worrying.” The scores from the seven items were aggregated to construct a composite index of anxiety symptoms (Cronbach’s *α* = 0.901), with higher scores indicating more severe anxiety symptoms.

Depressive symptoms were measured using the 9-item Patient Health Questionnaire (PHQ-9) (82), another self-reporting screening scale which has been validated in China (83). Participants reported depressive symptoms over the past 2 weeks using a 4-point scale ranging from 1 (“*not at all*”) to 4 (“*nearly every day*”). Sample items included “feeling down, depressed, or hopeless.” A composite depressive score was computed by summing up the scores of all nine items (Cronbach’s *α* = 0.884), with higher scores indicating more severe depressive symptoms.

#### 2.2.5 Physical exercise

Self-reported physical exercise was evaluated using the Godin and Shephard Leisure–Time Physical Activity Scale (GSLTPAS) (84). The GSLTPAS queried participants about their physical activity levels over the past 7 days, specifically asking, “Over the last 7 days, how many times on average did you engage in the following types of exercise for more than 15 min during your leisure time?” Participants classified the frequency of their exercise as “mild (minimal effort),” “moderate (not exhausting),” or “strenuous (heart beats rapidly).” The physical exercise index was determined by multiplying the frequency score by the corresponding metabolic equivalent (MET) value for each intensity level (i.e., light, moderate, and strenuous intensities corresponded to MET values of 3, 5, and 9, respectively) (84). Previous research has established the temporal stability of the GSLTPAS, with test–retest assessments conducted over 15 days (*k* = 0.65) and 30 days (*k* = 0.45) respectively, utilizing the kappa index (85).

### 2.3 Analytic approach

All statistical analyses were conducted using IBM SPSS 26.0 and Mplus 8.3. The statistical significance level was set at a two-tailed 0.05. Hypothesized mediating and moderating effects were examined using structural equation modeling (SEM). First, descriptive statistics were calculated for demographic characteristics. Second, the direct effects of expectations regarding school on emotional distress were assessed. Third, a parallel mediation model was constructed to explore mediating effects, utilizing expectations regarding school as the independent variable, and school exclusion and school acceptance as mediators, with latent emotional distress as the dependent variable. Finally, a moderated mediation model was developed to examine the interaction between exercise and school belongingness on emotional distress, and any resultant changes in mediating effects. Demographic variables were controlled for as covariates in all models. Model fit was evaluated using the chi-squared-degree of freedom ratio (*χ*^2^*/df*), comparative fit index (CFI), Tucker–Lewis index (TLI), root mean square error of approximation (RMSEA), and standardized root mean residual (SRMR) (86). Acceptable model fit was determined based on the following criteria: CFI > 0.90, TLI > 0.90, RMSEA <0.08, and SRMR <0.08 (87).

## 3 Results

### 3.1 Sociodemographic characteristics and correlations

The majority of the 1,019 participants (*M_age_* = 19.97 years) were male (50.6%), Han ethnic (88.9%), non-only child (64.3%) and undergraduate students (95.1%). A total of 173 (83.0%) participants were born in Xinjiang, indicating that they attended university in their born-province (see Table 1).

**Table 1:** Sociodemographic characteristics of the sample (*N* = 1,019).

Variable	Mean or *n* (%)
Age	19.97
Sex	
Male	516 (50.64%)
Female	503 (49.36%)
Ethnicity	
Han	906 (88.91%)
Others	113 (11.09%)
Birthplace	
Western of China	173 (16.98%)
Others	846 (83.02%)
Only child	
Yes	364 (35.72%)
No	655 (64.28%)
Education level	
Undergraduate	969 (95.09%)
Undergraduate first year	362 (35.53%)
Undergraduate second year	276 (27.09%)
Undergraduate third year	206 (20.22%)
Undergraduate fourth year or above	125 (12.27%)
Graduate	50 (4.91%)

**Figure 1:**
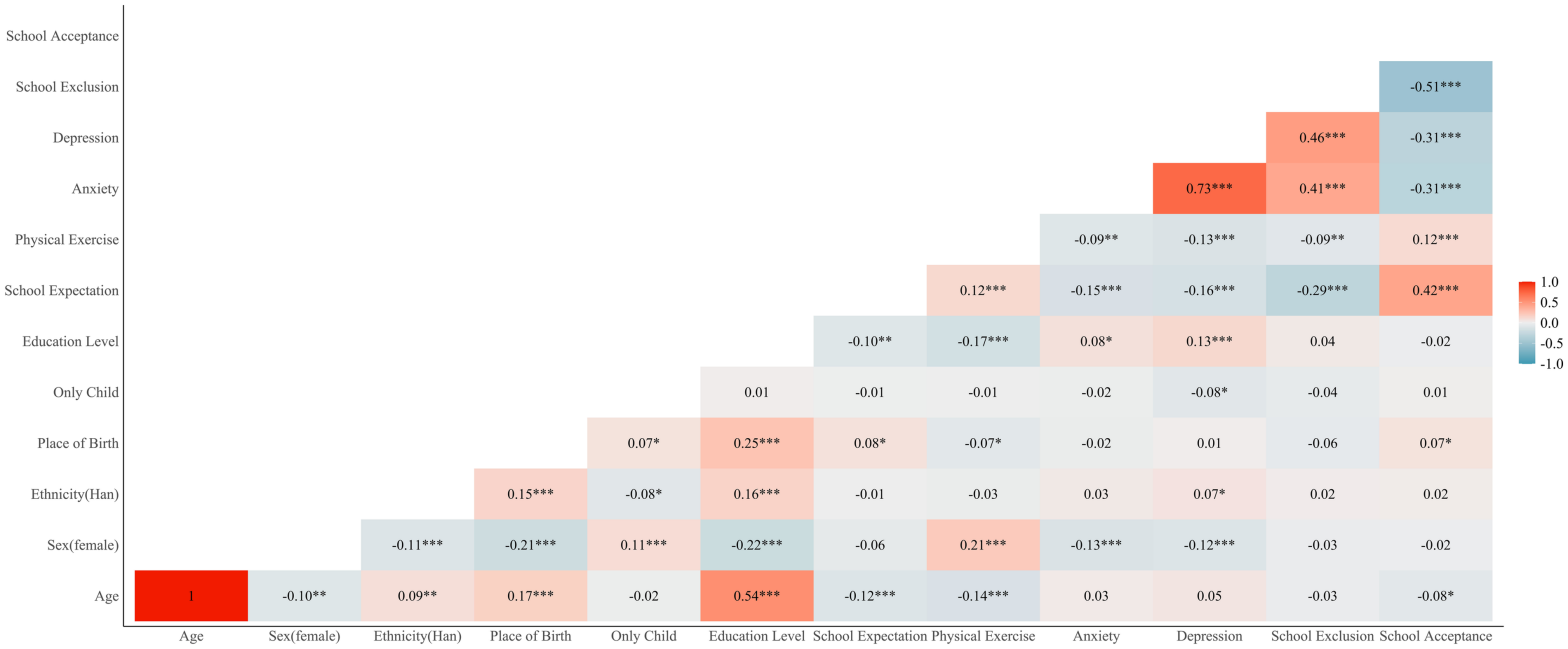
Pearson correlations between main variables. Sex, ethnicity, place of birth and only child were coded as dummy variables (i.e., male = 1, female = 0; Han ethnic group = 1, others = 0; Xinjiang = 1, others = 0). Education level is treated as a continuous variable, coded as 1–5, from undergraduate first year to graduate. *p < 0.05, **p < 0.01, ***p < 0.001.

The results of the correlation analysis (see Figure 1) indicated that expectations regarding school was negatively correlated with anxiety (*r* = −0.15, *p* < 0.001) and depression (*r* = −0.16, *p* < 0.001).

### 3.2 The direct effects of expectations regarding school

The direct effects of expectations regarding school on emotional distress were examined using a model where expectations regarding school served as the independent variable and emotional distress (i.e., anxiety and depressive symptoms) as the latent dependent variable. This model demonstrated a good fit with the data (χ^2^/*df* = 1.323, CFI = 0.999, TLI = 0.997, RMSEA = 0.018, 95% CI = [0.000, 0.067], SRMR = 0.007). The results revealed a negative association between expectations regarding school and emotional distress (*β* = −0.184, 95% CI = [−0.262, −0.112], *p* < 0.001).

### 3.3 The mediating effects of school belongingness

**Figure 2:**
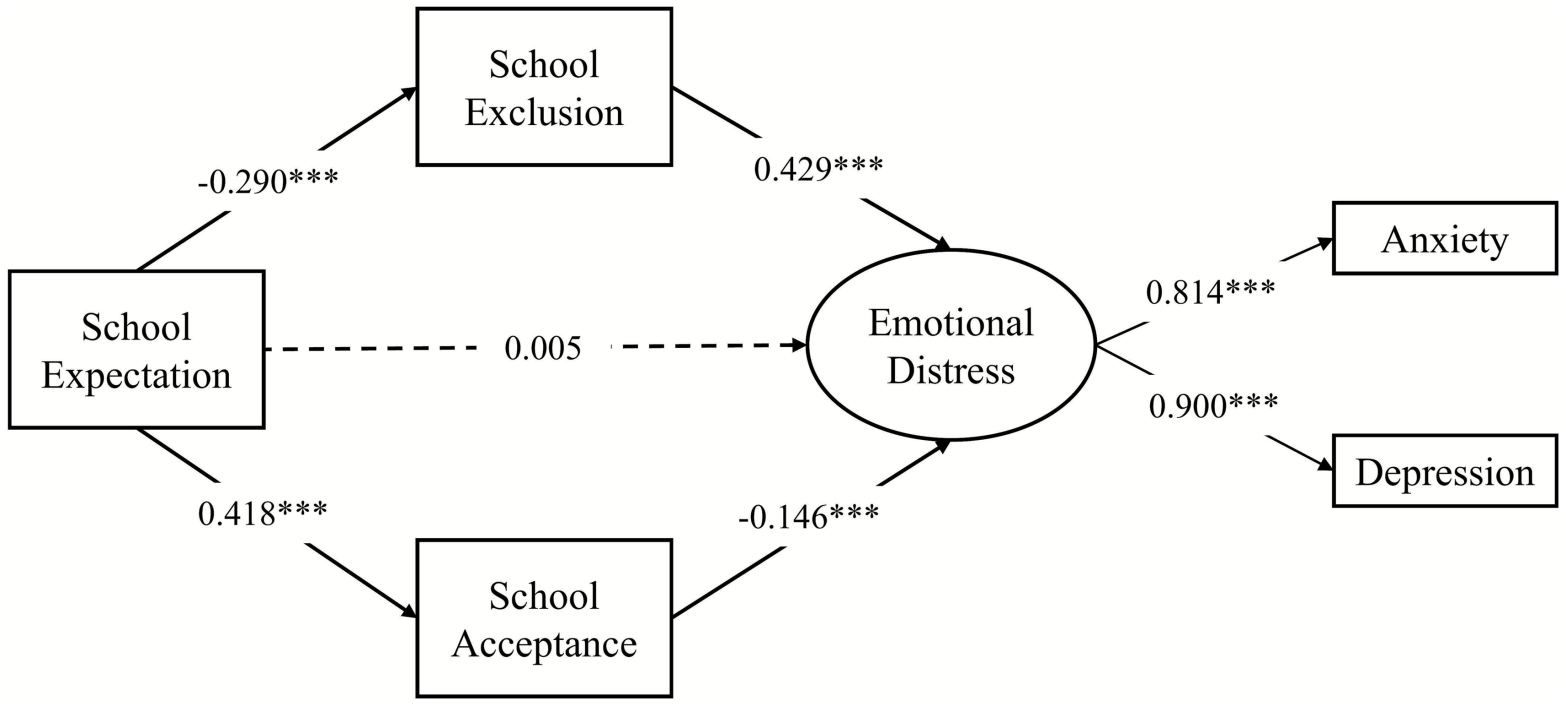
The mediation model. School exclusion and school acceptance were related in model calculation process. ***p < 0.001.

The mediating effects of school belongingness were examined through a parallel mediation model (see Figure 2), where two mediating variables were the two sub-dimensions of the school belongingness scale: school exclusion and school acceptance. The parallel mediation model evinced a good fit with the data (*χ*^2^*/df* = 1.353, CFI = 0.998, TLI = 0.996, RMSEA = 0.019, 95% CI = [0.000, 0.044], SRMR = 0.015). The results evidenced that expectations regarding school was negatively associate with school exclusion (*β* = −0.290, 95% CI = [−0.359, −0.224], *p* < 0.001), which in turn projected less emotional distress (*β* = 0.429, 95% CI = [0.349, 0.502], *p* < 0.001). Correspondingly, expectations regarding school was positively associated with school acceptance (*β* = 0.418, 95% CI = [0.353, 0.477], *p* < 0.001), which projected lower emotional distress (*β* = −0.146, 95% CI = [−0.223, −0.074], *p* < 0.001). Notably, after including the mediating variables, the direct effects became nonsignificant (*β* = 0.005, 95% CI = [−0.072, 0.076], *p* = 0.900). In summary, the results of the indirect effects demonstrated that both school exclusion and school acceptance significantly mediated the effects of expectations regarding school on emotional distress (see Table 2).

**Table 2:** The mediating effects of expectations regarding school on emotional distress.

Path	Mediating effects	Boot lower 95% CI	Boot upper 95% CI
Expectations regarding school → emotional distress	−0.19***	−0.23	−0.13
Expectations regarding school → school exclusion → emotional distress	−0.13***	−0.16	−0.09
Expectations regarding school → school acceptance → emotional distress	−0.06***	−0.10	−0.02

***p < 0.001.

### 3.4 The moderating effects of physical exercise

**Figure 3:**
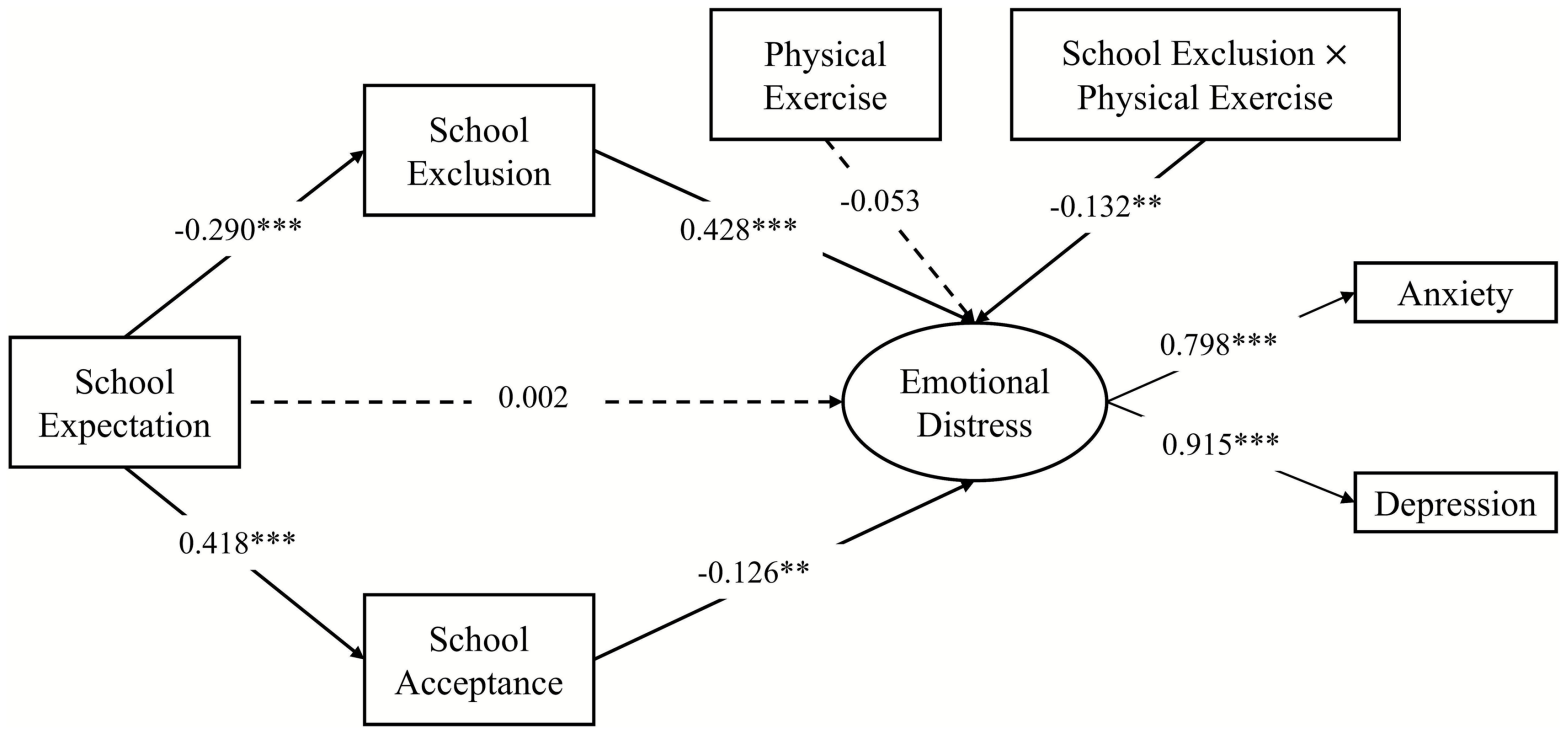
The moderated mediation model. School exclusion and school acceptance were related in model calculation process. **p < 0.01, ***p < 0.001.

The moderating effects of physical exercise were examined using a moderated mediation model (see Figure 3). Since the mediation model was a fully mediation model, the moderation of the direct effects was not considered. This model incorporated the interactions of physical exercise and school exclusion on emotional distress, and demonstrated a good fit (χ^2^/*df* = 2.441, CFI = 0.988, TLI = 0.977, RMSEA = 0.038, 95% CI = [0.022, 0.054], SRMR = 0.029). The results revealed that physical exercise moderated the mediating effects of school exclusion (see Figure 3), as indicated by the significant interaction between school exclusion and physical exercise on emotional distress (*β* = −0.132, 95% CI = [−0.220, −0.059], *p* < 0.01).

**Figure 4:**
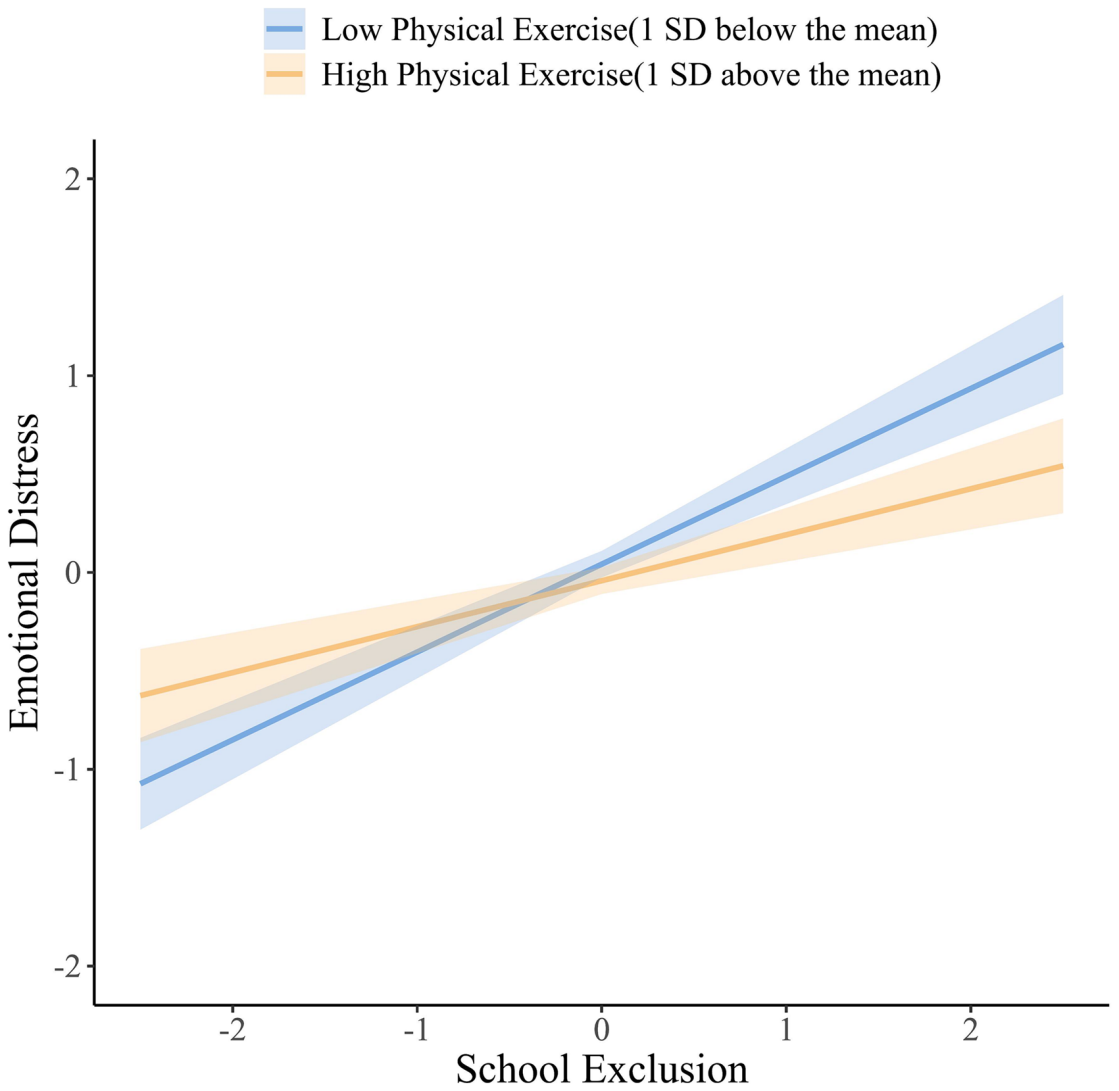
The simple slope for the moderating effects of physical exercise.

Specifically, Figure 4 illustrates that higher levels of school exclusion were associated with more severe emotional distress (*β* = 0.446, 95% CI = [0.359, 0.542], *p* < 0.001) when physical exercise was at a low level (1 *SD* below the mean). However, the relationship between school exclusion and emotional distress decreased at a high level (1 *SD* above the mean) of physical exercise (*β* = 0.234, 95% CI = [0.130, 0.331], *p* < 0.001).

Moreover, differences between the mediating effects of school exclusion were analyzed at different levels of physical exercise. The indirect effect of expectations regarding school on emotional distress through school exclusion at a high level of physical exercise (*β* = −0.068, 95% CI = [−0.104, −0.037], *p* < 0.001) was weaker than that at a low level of physical exercise (*β* = −0.130, 95% CI = [−0.171, −0.095], *p* < 0.001). These results indicate that the mediating effects of school exclusion between expectations regarding school and emotional distress diminish with an increase in physical exercise.

The present study also examined the moderating effect of exercise on the mediating effect of school acceptance by incorporating the interaction between physical exercise and school acceptance into this model. The results indicated that the moderating effect of physical exercise was not significant, as the interaction between school acceptance and physical exercise on emotional distress was not significant (*β* = 0.064, 95% CI = [−0.030, 0.156], *p* = 0.183).

## 4 Discussion

This study represents the first investigation into the effects of expectations regarding school on the emotional distress of college students, particularly within Western colleges of China. It delved into the relationships between expectations regarding school and emotional distress, with a specific emphasis on the roles of school belongingness and physical exercise.

Previous research on the relationship between expectations and student mental health has yielded conflicting conclusions (88, 89). However, this study provides evidence suggesting that expectations regarding school prior to enrollment can reduce the likelihood of emotional distress among Western college students. A possible explanation for this finding is that the school expectation of college students in Western China, who may have lower enrollment scores (90) or face poorer environments compared to those in Eastern China, could foster a greater sense of resilience as a protective factor in such circumstances (65). This positive psychological resource, along with psychological capital, assists them in coping with the stress of adaptation and transition resulting from a lack of material, economic, and intellectual resources (25, 29).

As hypothesized, school belongingness fully mediated the relationship between expectations regarding school and emotional distress, indicating that high expectations of college life may motivate students to transition better into the school environment, thereby reducing the risk of emotional distress. This phenomenon can be attributed to the fact that, for college students in Western China confronted with disadvantageous circumstances, higher expectations of college lives may lead to greater motivation to achieve their goals (42). This motivation is accompanied by a positive attention bias towards interpersonal cues (91) and proactive efforts to integrate into campus environments (41), ultimately enhancing the sense of belonging, which is positively associated with mental health (53). On the other hand, due to the uncertainty and unfamiliarity of the environment in Western China (with expectations potentially amplifying these feelings), students initiate familiarity-seeking behaviors (92). In university, this translates to seeking a sense of belonging, which reduces emotional distress. Moreover, the present study found that the mediating effect of school exclusion was higher than that of school acceptance in the parallel mediation model, consistent with previous findings (51). This result suggests that the positive effect of expectations regarding school on emotional distress is primarily achieved by reducing the sense of exclusion. Consequently, interventions targeting the sense of exclusion may be more effective than those targeting the sense of acceptance.

Within the context of expectation and belongingness, the current study underscores the significance of physical exercise on the mental health of college students, as supported by prior research (66). Physical exercise significantly moderated the effects of school exclusion on emotional distress in college students. Specifically, the levels of depression and anxiety due to exclusion were somewhat lower for students who were more physically active. However, the effects of school acceptance on emotional distress were not moderated by physical exercise. Correspondingly, the mediating effects of exclusion in the relationship between expectations regarding school and emotional distress were moderated by physical exercise, whereas the mediating effect of acceptance was not. In addition to the effects of physical exercise on self-esteem, self-efficacy, and brain structure and function that we previously mentioned, there may be other physiological and social psychological mechanisms at play. Physical exercise effectively reduces inflammation and promotes neuroplasticity (93). This, in turn, aids adaptive stress responses and enhances psychological resilience (94), helping to mitigate the emotional distress caused by the sense of exclusion. The social psychological mechanisms through which physical exercise exerts its effects may also manifest in promoting social interaction (95), and increasing social support (96). In summary, engaging in physical exercise on a weekly basis can play a protective role in weakening the detrimental effects of low expectations and high exclusion on mental health.

This study carries both theoretical and practical implications. This study makes a theoretical contribution by introducing the concept of “expectations regarding school.” As a novel construct, expectations regarding school encompasses students’ pre-university expectations about various aspects of their academic and campus life. By shifting the focus from traditional academic expectations to a more holistic understanding of student expectations, this research provides a fresh perspective on how these expectations serve as psychological resources, promoting school belongness and mental health, offering a framework that can be expanded in future studies. Additionally, this study is the first to evaluate the moderating effect of physical exercise on these processes, revealing its protective effect on excluded college students. This theoretical framework could be extended to other regions and cultures that share similar socio-economic constraints. For example, the resilience demonstrated by students in Western China might also be observed in other regions with limited resources, such as rural areas in developing countries. This expands the existing literature on psychological capital and resilience, suggesting that positive expectations can mitigate the adverse effects of socio-economic challenges across diverse settings. Furthermore, by innovatively integrating environmental, physical, and mental factors, the study enriches the perspective of socioecological psychology, which focuses on how individuals respond and adapt to interpersonal, economic, and political environments.

In terms of practical significance, the findings offer valuable insights for policymakers, administrators, and mental health educators in Western China’s colleges. Firstly, for western university students facing objectively disadvantaged environments, universities can help students establish positive expectations through recruitment campaigns, orientation education, and other activities. Additionally, fostering a sense of belonging on campus through enhancing campus atmosphere and peer support can further contribute to the mental health. Secondly, physical exercise is also a relatively easy intervention to implement. Effective measures include investing in sport resources, offering courses, organizing group sports activities, and utilizing sports apps to track students’ exercise data for rewards and incentives. However, in practical interventions, there may be certain limitations in implementation. Students in universities of Western China may differ in terms of academic levels, economic status, cultural backgrounds, and geographical environments. Therefore, the intervention measures need to be tailored accordingly. For example, students’ expectations of the university may be influenced by the prosperity and natural environment of the region where the university is located, factors that universities cannot change. Additionally, the implementation of intervention measures such as promoting physical exercise may be hindered by factors like facilities, teaching staff, and funding. Moreover, ensuring the long-term effectiveness of intervention measures in the face of external pressures is also a critical consideration. Lastly, the above considerations and insights on the significance of these practices can also be extended to other regions and cultures, especially in underdeveloped or rural areas where access to mental health resources is limited.

The study acknowledges several limitations. Firstly, although the self-developed measurement of expectations regarding school employed in this study exhibits good reliability and validity, it may not necessarily constitute a rigorous measurement tool, nor can it be guaranteed to possess universal applicability. Future research should develop a more comprehensive and multidimensional scale to better capture students’ expectations regarding school. Secondly, relying heavily on self-report measures for assessing emotional distress and physical activity can be subjective and susceptible to bias. This could be addressed by employing a more diverse research paradigm, such as immune and metabolic function markers, brain imaging, and experimental manipulation, which could provide further insights into the role of physical exercise in mental health. Accordingly, these approaches can also be used to explore more direct factors that protect the mental health of excluded students, such as mindfulness (97), physiological hormones (69), and cognitive function during exercise (75). Thirdly, although the sample included students from various universities in Xinjiang and represented a diverse range of demographics, convenience sampling and snowball sampling inherently carries the risk of selection bias. Specifically, the participants who were accessible and willing to participate in the study might differ in significant ways from those who were not. As a result, the findings may not capture the experiences of students in more remote areas or those with limited access to university resources, who may actually align more closely with the aims of our research. Therefore, caution should be exercised when generalizing the results to the broader population of college students in Western China. Fourthly, these findings do not establish a causal relationship due to the cross-sectional design, as it relies on students’ retrospective reports of their pre-enrollment expectations while they are already enrolled in university. Future research should consider employing longitudinal designs that span both before and after a student’s enrollment, which can ensure the accurate measurement of pre-enrollment expectations and provide more robust evidence of causal relationships. Experimental studies of the expectations regarding school, school belongingness and physical exercise could also help in understanding the causal impact on emotional distress.

Several promising directions for future research are worth exploring. Firstly, many variables may influence expectations regarding school. For example, a student attending the same university as one or both of their parents would likely have very different expectations than a first-generation student. Thus, Future research should more comprehensively cover these potential influencing factors, such as student background, family dynamics, and peer relationships (16, 18). Moreover, potential gender and ethnicity differences in how expectations regarding school and physical exercise influence emotional distress merit attention in future research. The results revealed that there was no significant correlation between expectations regarding school, school belongingness, and gender. However, male students reported higher levels of physical activity and fewer emotional distresses (see Figure 1). Previous studies have shown that male and female students often experience and cope with stress differently, and these differences could affect how interventions are designed and implemented (98). For example, males may respond more to physical interventions like exercise, while females might benefit more from social and psychological support systems. A deeper exploration of these gender-specific dynamics would enable more targeted interventions to improve student mental health. Similarly, the present study’s sample consisted primarily of Han Chinese students, but given the ethnic diversity within Western China, many ethnic minority groups may face unique cultural and educational challenges. Understanding these could provide more culturally sensitive and effective interventions for reducing emotional distress among minority students. Additionally, cultural context should be emphasized to distinguish how emotional distress is influenced among university students from different cultures. In cultures rooted in holistic thinking and collectivism, the results could highlight the importance of social relationships and collective support (99). School belongingness and group-oriented physical activities may play a more significant role in reducing emotional distress. Conversely, in cultures characterized by analytical thinking and individualism, the effect of school belongingness may be weakened and physical exercise might more directly impact emotion regulation rather than acting as a moderating role for school exclusion. These aspects deserve further validation in future research. Furthermore, regarding the moderating and intervention effects of physical exercise, future research may focus on distinguishing differences in the effects of aerobic and anaerobic exercise (71) or between team sports and individual sports (67) to better assist practitioners in designing campus physical exercise programs to enhance students’ physical and mental health. Finally, future research should explore the impact and mechanisms of expectations regarding school on emotional distress in more economically developed regions to determine whether similar results would be obtained or not.

In conclusion, this study is a pioneering effort in identifying a positive correlation between heightened expectation for future college or university enrollment in Western China and students’ enhanced sense of belonging to school. Consequently, this amplifies the manifestation role of emotional distress among students. Moreover, it underscores the validation of the buffering role of physical exercise for school exclusion, albeit not for fostering school acceptance. The promotion of positive exercise routines among college students emerges as a pivotal strategy in shielding against the adverse effects of challenges encountered in assimilating into the campus environment, thereby curbing anxiety and depressive symptoms.

## Data Availability

The datasets, codes and materials used in the present study are available from the corresponding author on reasonable request.
