# Supplementary material for: Expectations regarding school decreases emotional distress among college students in Western China: the buffering role of physical exercises
Source: Front Public Health. 2024 Nov 6;12:1412199. doi: 10.3389/fpubh.2024.1412199 (PMC11576314; doi:10.3389/fpubh.2024.1412199)

Supplementary Material

# Psychometric properties of the expectations regarding school questionnaire

## Aim of this study

This study aims to investigate the psychometric properties of the four-item self-designed questionnaire of students’ expectations regarding school.

## Method

### *Participants and procedure*

In this study, the same dataset was used in the main body of the paper, consisting of a total of 1,019 participants. The participants were randomly divided into two samples using a 0-1 uniform random variable generated by SPSS 26.0. About 50% of the participants (i.e., those with a generated random number less than or equal to 0.5) were assigned into sample 1 (*N*_1_ = 508), and the remaining participants were allocated to sample 2 (*N*_2_ = 511).

Expectations regarding school

As mentioned in the “Materials” section of the main body of this paper, students’ expectations regarding school was measured using four self-designed questions (see detailed items in Table S2).

Analytic approach

All statistical analyses were conducted using IBM SPSS 26.0 and Mplus 8.3. The statistical significance level was set at a two-tailed 0.05. First, a series of t-test and chi-squared test were conducted on demographic variables to prove that there were no significant differences between the two samples.

Next, the validity of the measurement was examined by EFA and CFA. Specifically, first, an exploratory factor analysis (EFA) was utilized on sample 1 to explore the factor structure of the questionnaire, using principal component analysis and direct oblimin rotation. The criterion of eigenvalues greater than 1 was adopted in principal component analysis. A factor loading of 0.3 was used as a cut-off above which an item was viewed as a reliable indicator of that factor. Second, a confirmatory factor analysis (CFA) was performed on sample 2 by maximum likelihood estimates to confirm the models derived from EFA. Model fit was evaluated using the chi-squared-degree of freedom ratio (*χ2/df*), comparative fit index (CFI), Tucker–Lewis index (TLI), root mean square error of approximation (RMSEA), and standardized root mean residual (SRMR) (1). Acceptable model fit was determined based on the following criteria: CFI > 0.90, TLI > 0.90, RMSEA < 0.08, and SRMR < 0.08 (2).

In addition, Cronbach’s alpha was calculated to check the reliability of the measurement of students’ expectations regarding school on sample 2, with an alpha greater than 0.80 indicating a good fit and greater than 0.90 indicating excellent internal consistency reliability.

## Results

Demographic characteristics and comparisons between two samples

The majority of the 508 participants in sample 1 (*M_age_* = 19.94 years) were male (52.17%), Han ethnic (89.17%) and non-only child (66.34%). In the sample 2, participants were largely of female (50.88%) and Han ethnicity (88.65%) and non-only child (62.23%). In both samples, less than 20% of the students were born in Xinjiang (16.34% in sample 1 and 17.61% in sample2). In general, the two samples exhibited similar demographic characteristics (*see* Table S3).

The t-test results indicated no statistically significant differences between two random samples on age and educational level (*p* > 0.05). The chi-squared test results showed that other demographic variables, including sex, ethnicity, place of birth and whether participants were an only child, were not statistically associated between the two samples (*p* > 0.05).

Construct validity

Results of the Kaiser-Meyer-Olkin (KMO) measure of sampling adequacy test and the Bartlett test of sphericity revealed that sample 1 was suitable for EFA (KMO = 0.782, *χ2* = 942.848, *df* = 6, *p* < 0.001). Utilizing principal component analysis, only one factor was exacted, accounting for 69.85% of the total variance. Factor loadings for all four items were larger than 0.3, and the community was larger than 0.50 (3) (*see* Table S4). In addition, the one-factor structure was consistent with the connotation of expectations regarding school. Thus, the one-factor structure was adopted.

To further confirm the one-factor structure, we performed CFA with the maximum likelihood method, using the other half sample. A single-factor model was built (*see* Figure S1), which showed good fit (*χ2/df* = 1.563, CFI = 0.999, TLI = 0.996, RMSEA = 0.033, 90% CI = [0.000, 0.128], SRMR = 0.006).

Reliability

The reliability of the measurement was proved to be good (Cronbach’s *α* = 0.848). After deleting individual items, the Cronbach's alpha coefficient decreased, indicating that each item contributed well to the internal consistency of the scale (*see* Table S4).

## Conclusion

In conclusion, the questionnaire of students’ expectations regarding school showed high reliability with the one-factor structure, consistent with the connotation of this variable. Thus, the four questions formed a useful measurement, which could promote both the assessment and research of students’ expectations regarding school in the Chinese population. It could be an effective tool for educators to explore students' expectations regarding the overall school environment.

## References

1. Hu L-t, Bentler PM. Fit Indices in Covariance Structure Modeling: Sensitivity to Underparameterized Model Misspecification. *Psychological methods* (1998) 3(4):424.

2. Wen Z, Hau K-T, Herbert WM. Structural Equation Model Testing: Cutoff Criteria for Goodness of Fit Indices and Chi-Square Test. *Acta psychologica sinica* (2004) 36(02):186.

3. Fabrigar LR, Wegener DT. *Exploratory Factor Analysis*: Oxford University Press (2012).

# Supplementary Figures and Tables

| Variables | Measurement | Abbreviation | Item | Scoring | Reliability |
| --- | --- | --- | --- | --- | --- |
| Expectations Regarding School | Self-designed questions | —— | 4 | 5-point Likert | *α* = 0.848 |
| School Belongingness | School Belongingness Scale | SBS | 10 | 4-point Likert | *α* = 0.871 |
| School Exclusion | School Exclusion Scale | SES | 5 | 4-point Likert | *α* = 0.832 |
| School Acceptance | School Acceptance Scale | SAS | 5 | 4-point Likert | *α* = 0.858 |
| Emotional Distress |  |  |  |  |  |
| Anxiety | Generalized Anxiety Disorder Scale | GAD-7 | 7 | 4-point Likert | *α* = 0.901 |
| Depression | Patient Health Questionnaire | PHQ-9 | 9 | 4-point Likert | *α* = 0.884 |
| Physical Exercise | Godin and Shephard Leisure–Time Physical Activity Scale | GSLTPAS | 3 | Weighted by MET values | *k* = 0.65 (15 days) and *k* = 0.45 (30 days) in previous research |

**Table S1.** Measurement for all main variables

|  | The following questions are about your expectations of campus life. Please try to recall your thoughts before entering university and select the number that corresponds to the degree of your expectations: | |
| --- | --- | --- |
|  | 1. How were your expectations of campus environment? | *(1) Very low (2) Relatively low (3) Average (4) Relatively high (5) Very high* |
|  | 2. How were your expectations of campus life? | *(1) Very low (2) Relatively low (3) Average (4) Relatively high (5) Very high* |
|  | 3. How were your expectations of academic experience? | *(1) Very low (2) Relatively low (3) Average (4) Relatively high (5) Very high* |
|  | 4. How were your expectations of extracurricular activities? | *(1) Very low (2) Relatively low (3) Average (4) Relatively high (5) Very high* |

**Table S2.** The four-item questionnaire for measuring students’ expectations regarding school

| Variables | Mean / *n* (%) | | |  | *t* / *χ2* | *p* |
| --- | --- | --- | --- | --- | --- | --- |
|  | **Total (*n* = 1019)** | **Sample 1 (*N*_1_ = 508)** | **Sample 2 (*N*_2_ = 511)** |  |  |  |
| Age | 19.97 | 19.94 | 20.00 |  | -0.273 | 0.354 |
| Educational Level | 2.31 | 2.36 | 2.26 |  | 1.137 | 0.256 |
| Sex |  |  |  |  | 0.946 | 0.331 |
| Male | 516 (50.64%) | 265 (52.17%) | 251 (49.12%) |  |  |  |
| Female | 503 (49.36%) | 243 (47.83%) | 260 (50.88%) |  |  |  |
| Ethnicity |  |  |  |  | 0.071 | 0.790 |
| Han | 906 (88.91%) | 453 (89.17%) | 453 (88.65%) |  |  |  |
| Others | 113 (11.09%) | 55 (10.82%) | 58 (11.35%) |  |  |  |
| Birthplace |  |  |  |  | 0.293 | 0.588 |
| Western China | 173 (16.98%) | 83 (16.34%) | 90 (17.61%) |  |  |  |
| Others | 846 (83.02%) | 425 (83.66%) | 421 (82.39%) |  |  |  |
| Only Child |  |  |  |  | 1.872 | 0.171 |
| Yes | 364 (35.72%) | 171 (33.66%) | 193 (37.77%) |  |  |  |
| No | 655 (64.28%) | 337 (66.34%) | 318 (62.23%) |  |  |  |

**Table S3.** Demographic characteristics and between-group difference tests of the two samples

| Items | Mean ± SD | Factor 1 | Communality | Cronbach’s *α* if item deleted |
| --- | --- | --- | --- | --- |
| 2. How were your expectations of campus life? | 3.67 ± 0.82 | 0.892 | 0.796 | 0.829 |
| 3. How were your expectations of academic experience? | 3.64 ± 0.83 | 0.848 | 0.719 | 0.774 |
| 1. How were your expectations of campus environment? | 3.70 ± 0.83 | 0.801 | 0.642 | 0.802 |
| 4. How were your expectations of extracurricular activities? | 3.55 ± 0.93 | 0.797 | 0.636 | 0.823 |

**Table S4.** Factor loadings and reliability of the questionnaire of expectations regarding school (*N* = 508)

**Figure S1.** The one-factor structure of the questionnaire of expectations regarding school


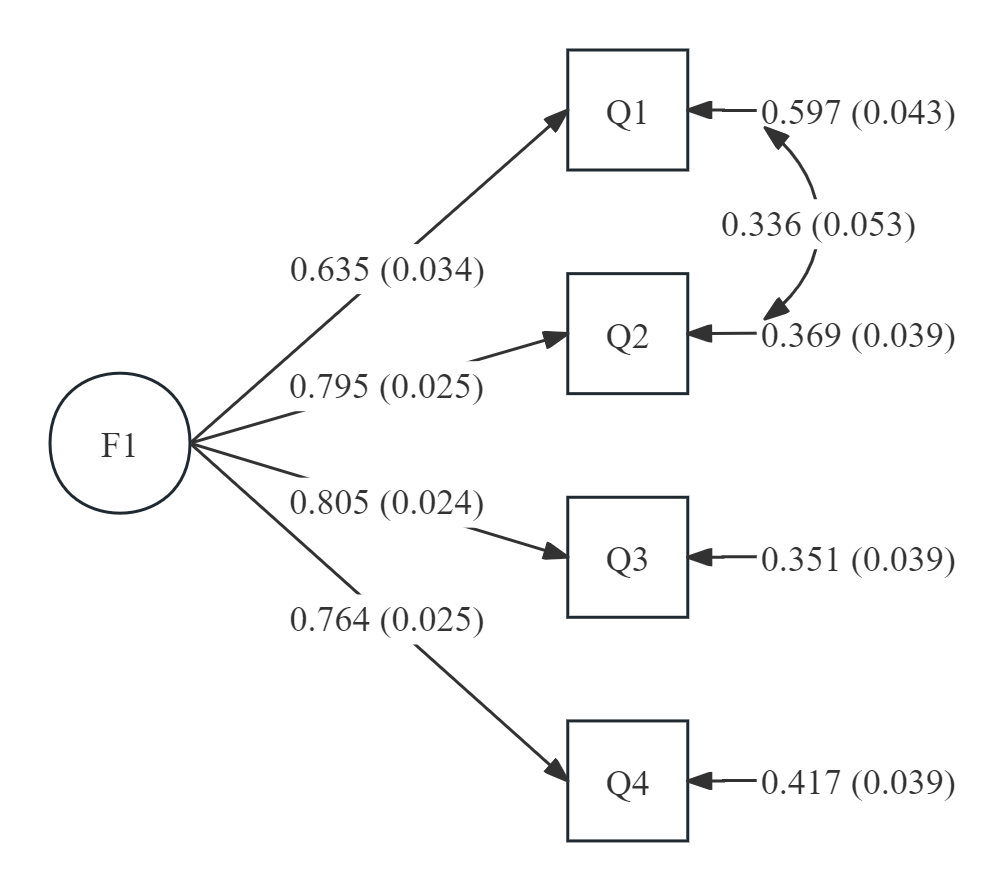

Supplement: Supplementary file 1 [file Data_Sheet_1.docx]
